# Evaluation of Aniseikonia in Patients with Successfully Treated Anisometropic Amblyopia Using Spatial Aniseikonia Test

**DOI:** 10.3390/jcm12113766

**Published:** 2023-05-30

**Authors:** Ryusei Takigawa, Kakeru Sasaki, Masakazu Hirota, Maki Nakagawa, Kozue Sasaki, Toshifumi Mihashi, Yoshinobu Mizuno, Atsushi Mizota, Kumiko Matsuoka

**Affiliations:** 1 Division of Orthoptics, Graduate School of Medical Care and Technology, Teikyo University, Itabashi, Tokyo 173-8605, Japan; 2 Department of Orthoptics, Faculty of Medical Technology, Teikyo University, Itabashi, Tokyo 173-8605, Japan; ssk-kkr@med.teikyo-u.ac.jp (K.S.); hirota.ortho@med.teikyo-u.ac.jp (M.H.); mihashi.toshifumi.gt@med.teikyo-u.ac.jp (T.M.); shiro-mm@med.teikyo-u.ac.jp (K.M.); 3 Department of Ophthalmology, School of Medicine, Teikyo University, Itabashi, Tokyo 173-8605, Japan; mknkgw@med.teikyo-u.ac.jp (M.N.); tama-0518@med.teikyo-u.ac.jp (K.S.); bfold0123@gmail.com (Y.M.); mizota-a@med.teikyo-u.ac.jp (A.M.); 4 Nishikasai Inouye Eye Hospital, Edogawa-Ku, Tokyo 134-0088, Japan

**Keywords:** aniseikonia, anisometropia, anisometropic amblyopia, amblyopia, binocular vision

## Abstract

Anisometropic amblyopia is decreased visual acuity in one eye, and treatment consists of wearing complete corrective spectacles. Aniseikonia occurs with complete correction of anisometropia using spectacles. Aniseikonia has been ignored when treating pediatric anisometropic amblyopia because of the prevailing belief that anisometropic symptoms are suppressed by adaptation. However, the conventional direct comparison method for evaluating aniseikonia significantly underestimates the degree of aniseikonia. This study investigated whether the adaptation occurs due to long-term anisometropic amblyopia treatment in patients who have had successful amblyopia treatment using a spatial aniseikonia test with high accuracy and repeatability compared with the conventional direct comparison method. The amount of aniseikonia was not significantly different between the patients with successful amblyopia treatment and individuals with anisometropia without a history of amblyopia. In both groups, the aniseikonia per 1.00 D of anisometropia and the aniseikonia per 1.00 mm of aniso-axial length were comparable. The repeatability of the amount of aniseikonia using the spatial aniseikonia test did not differ significantly between the two groups, indicating a high degree of agreement. These findings suggest that aniseikonia is not adapted to amblyopia treatment and that aniseikonia increases as the difference between spherical equivalent and axial length increases.

## 1. Introduction

Aniseikonia is a state of difference in the perceptual shape and/or images presented to the visual cortex via both eyes [1]. The prevalence of aniseikonia is approximately 7.8% [2], and an amount of aniseikonia of >2% induces ocular adverse effects [3], such as visual fatigue [4], headache [5,6], spatial distortion [6,7,8], reduction of stereoacuity [9,10,11], and macropsia or micropsia [12,13,14]. Aniseikonia is caused by various factors, including ocular refraction differences between the left and right eye [1], retinal disease [13,15,16], and differences in cortical neural processing [17]. In clinical practice, aniseikonia relates to anisometropia, in which the ocular refraction is different in the two eyes because the spectacle magnifications are different between the right and left lenses in fully corrected spectacles [2]. In anisometropia in particular, the difference in ocular refraction between both hyperopic eyes is >2.00 D, which can lead to amblyopia (anisometropic amblyopia) in one eye if left unchecked [3,18]. Although there are risks of ocular adverse effects associated with aniseikonia, the most common treatment is the use of best-corrected spectacles [19].

Previously, patients with anisometropic amblyopia hardly complained of anisometropic symptoms because of the effects of adaptation and suppression in the cerebral cortex [3]. South et al. reported that patients with anisometropic amblyopia perceived aniseikonia with significant variety; the amount of aniseikonia also varied significantly [20]. This result can be considered to have two causes. First, adaptation and suppression in the visual cortex varied the amount of aniseikonia. Second, the accuracy and reproducibility of the aniseikonia test varied. There are two methods for measuring aniseikonia: direct comparison and space eikonometry. The direct comparison method separates the two eyes using red–green duochrome filters or other means and directly compares the image sizes of the left and right eyes. In the clinic, aniseikonia is usually evaluated using the direct comparison method [21,22,23]. However, this direct comparison method underestimates and modulates the degree of aniseikonia because of the influence of peripheral fusion and sensory adaptation, which cancel out the image differences caused by aniseikonia in binocular vision [24,25,26,27]. Moreover, the direct comparison method interprets aniseikonia as either a magnification or a constriction. However, aniseikonia is usually defined as the relative ratio of how significant the image of one eye is compared with the image of the other eye [1]. Therefore, it would be inappropriate to assume that the magnification and contraction of the visual target are identical. Space eikonometry detects the spatial distortion caused by aniseikonia as binocular disparity. Space eikonometry is considered the gold standard for the determination of aniseikonia because of its high measurement accuracy [28]. However, space eikonometry is complicated, requires the patient’s understanding, is measured up to aniseikonia of 5%, [29,30], and has a more significant variability in measurements. Therefore, space eikonometry has not been used in clinical practice [25].

**Figure 1:**
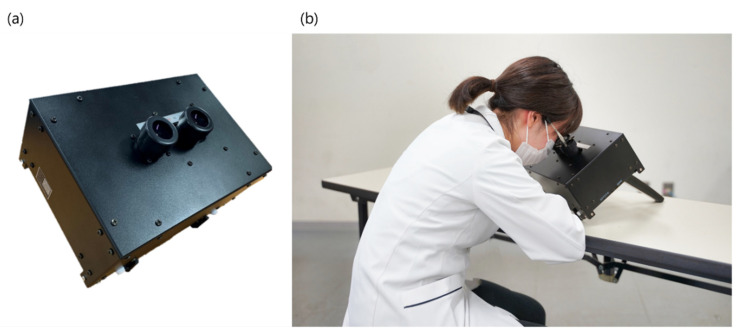
The spatial aniseikonia test (SAT). (**a**) The SAT has +8.00 D lenses built into the left and right barrels, and a monitor is built in at the focal point of the lens (125 mm). (**b**) Each subject viewed the targets displayed on the SAT under subjective best-corrected visual acuity with an optic lens.

The spatial aniseikonia test (SAT) is an aniseikonia testing system based on space eikonometry (Figure 1) [31]. Because the SAT uses an application in a personal computer for control, it is possible to measure the aniseikonia of the targets with no upper limit. In addition, the structure of the targets is simplified. This makes them easier to understand and measure for the subject. Sasaki et al. reported that the accuracy of the measurement of the degree of aniseikonia generated artificially with a size lens was significantly higher using the SAT vs. the direct comparison method in healthy individuals [32]. Therefore, we expected that the SAT could be used to assess the true amount of aniseikonia by reducing the variability in the patients with amblyopic aniseikonia. Furthermore, we hypothesized that the amounts of aniseikonia were not different between the anisometropic patients with amblyopia and without amblyopia.

As the first step in the verification of the hypothesis, this study aimed to determine whether anisometropic amblyopia treatment truly inhibits aniseikonia. Therefore, we evaluated the degree of aniseikonia between patients with successful anisometropic amblyopia treatment and anisometropia volunteers without a history of anisometropic amblyopia.

## 2. Materials and Methods

### 2.1. Subjects

In this study, the subjective spherical equivalent (SE) difference of ≥2.00 D between the right and left eyes was defined as anisometropia. Anisometropic amblyopia included astigmatism of <2.00 D, without strabismus, central fixation, and meeting the cure criteria for amblyopia treatment. The cure criterion for amblyopia treatment was defined as meeting at least one of the Pediatric Eye Disease and Investigator Group (PEDIG) criteria for successful amblyopia treatment, similar to the cure criteria for anisometropic amblyopia in previous studies [33,34,35,36].

(1) Interocular difference in acuity <0.2 logMAR.
(2) Best-corrected visual acuity improvement of 3 or more logMAR lines.
(3) Best-corrected visual acuity amblyopic eye visual acuity ≤0.1 logMAR.

Fourteen patients with anisometropic amblyopia (mean ± standard deviation, 8.2 ± 1.5 years), who initiated treatment between October 2011 and July 2021, were recruited from Teikyo University Hospital (Tokyo, Japan) as the amblyopic group in this study. Then, 16 volunteers (20.3 ± 1.7 years) without a history of amblyopia were included in the anisometropic group as a control. Astigmatism of >2.00 D and other diseases were excluded.

This study was conducted between August 2021 and November 2022. All patients and volunteers were measured for objective refraction (ARK-1; Nidek Inc., Aichi, Japan), subjective refraction at a test distance of 5 m, and axial length (AL) (OA-2000; Tomey Inc., Aichi, Japan), and stereoacuity was quantitatively assessed with the Titmus stereo test (Stereo Optical Co., Chicago, IL, USA) with graded circles under the best-corrected visual acuity. Stereoacuity was converted to the logarithm of an arcsecond (log arcsec). SE was measured based on objective refraction using 1% cyclopentolate (Alcon, Fort Worth, TX, USA).

After we explained the nature of the study and possible complications to the subjects, all subjects provided informed consent. This investigation adhered to the tenets of the World Medical Association Declaration of Helsinki. This study was approved by the Institutional Review Board of Teikyo University (Approval No. 20–161).

### 2.2. Evaluation of Aniseikonia

#### 2.2.1. Apparatus

**Figure 2:**
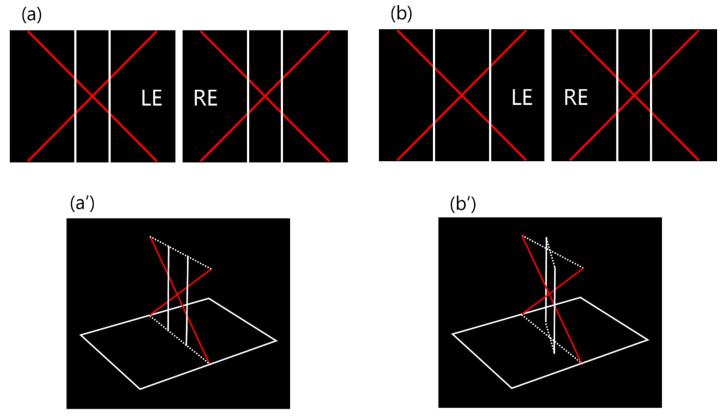
SAT target. (**a**) The target consists of an oblique red line and a white vertical line on a black background. (**b**) In aniseikonia, the retinal image of one eye is perceived as larger than that of the other eye, the target is enlarged, and the distance between the two white vertical lines is wider than that in the absence of aniseikonia. (**a′**) In the absence of aniseikonia, all lines appear parallel to the frontal plane. (**b′**) In aniseikonia, the eye with the enlarged retinal image perceives the white vertical line as being farther away than the other eye. For example, if the image presented to the left eye appears larger than that of the right, the white vertical line of the left eye appears to recede. SAT, spatial aniseikonia test; LE, left eye; RE, right eye.

The SAT has +8.00 D lenses built into the left and right barrels and a built-in monitor (10.1 Inch Portable Monitor; UPERFECT Inc., Shenzhen China) at the focal point of the lens (Figure 1a). When the subject looks through the barrels (Figure 1b), an oblique red line and a white vertical line appear on a black background (Figure 2a).

In the absence of aniseikonia, the two white vertical lines are perceived to be equidistant (Figure 2a). In aniseikonia, the retinal image of one eye is perceived as larger than that of the other eye; the target is enlarged, and the distance between the two white vertical lines is wider than that in the absence of aniseikonia (Figure 2b). As a result, the eye with an enlarged retinal image perceives the white vertical lines as farther apart than the other eye (Figure 2b).

#### 2.2.2. Measurement Procedure

Each subject viewed the targets displayed on the SAT under subjective best-corrected visual acuity with a spectacle lens. The target was started with a 10% magnification of the image in the right eye. The subjects were asked to relate the front–back relationship of the two white lines, and the magnification of the white line with higher magnification (larger magnification) was reduced. When the two white lines became the same in depth, the magnification was defined as aniseikonia. After examination of the right eye for aniseikonia, the left eye’s image was re-examined at 10% magnification. Examinations were always performed from the right eye to the left eye in that order. Three magnified values were obtained for the right eye and three for the left eye. Aniseikonia was defined as the mean value of a total of six values.

### 2.3. Data Analyses

The SE, AL, and cornea radius of curvature were calculated as absolute values of the differences between left and right eyes. The difference between left and right eyes of SE was defined as the difference of SE. The difference between left and right eyes of AL was defined as the difference of AL. The aniseikonia per 1.00 D of anisometropia and the aniseikonia per 1.00 mm of aniso-AL were calculated using the following formula:

▪ Aniseikonia per 1.00 D of anisometropia = aniseikonia/difference of SE.
▪ Aniseikonia per 1.00 mm of aniso-AL = aniseikonia/difference of AL.

Aniseikonia was defined as the difference in retinal magnification between both eyes.

### 2.4. Statistical Analyses

The amount of aniseikonia, aniseikonia per 1.00 D of anisometropia and 1.00 mm of aniso-AL, SE, AL, and corneal radius of curvature, stereoacuity, and visual acuity in the amblyopic and anisometropic groups were assessed for normality of the data by the Shapiro–Wilk test. For normal distributions, the homogeneity of variance was assessed by Levene’s test. Student’s *t*-test was performed if the variance was equal, and Welch’s *t*-test if the variance was not equal. The Mann–Whitney U test was performed for non-normal distributions.

The test–retest reliability of aniseikonia in the SAT was analyzed using intraclass correlation coefficients (ICC 1, 6).

The correlations between the difference of SE and the amount of aniseikonia, the difference of AL and the amount of aniseikonia, and the stereoacuity and the amount of aniseikonia in the two groups were analyzed by single regression analysis.

IBM SPSS Statistics version 28.0 (IBM Corp., Armonk, NY, USA) was used to determine the significance of the differences, and a *p*-value of <0.05 was considered significant.

## 3. Results

Aniseikonia had a normal distribution for both the amblyopic and anisometropic groups (amblyopic group, *p* = 0.083; anisometropic group, *p* = 0.24). The difference of SE was not normally distributed for the amblyopic group (*p* = 0.038) but was normally distributed for the anisometropic group (*p* = 0.35). Aniseikonia per 1.00 D of anisometropia followed a normal distribution for both the amblyopic and anisometropic groups (amblyopic group, *p* = 0.39; anisometropic group, *p* = 0.70). Aniseikonia per 1.00 mm of aniso-AL was not normally distributed for the amblyopic group (*p* = 0.005) but was normally distributed for the anisometropic group (*p* = 0.47). The difference of AL had a normal distribution for both the amblyopic and anisometropic groups (amblyopic group, *p* = 0.150; anisometropic group, *p* = 0.093). The corneal radius of curvature was not normally distributed for the amblyopic group (*p* = 0.004) but was normally distributed for the anisometropic group (*p* = 0.164). Stereoacuity was normal distribution for the amblyopic group (*p* = 0.24) but not for the anisometropic group (*p* = 0.023). Visual acuity was not normally distributed in both the amblyopic and anisometropic groups (amblyopic group, *p* = 0.004; anisometropic group, *p* < 0.001). Aniseikonia, aniseikonia per aniso-AL 1.00 mm, and difference of AL were equally distributed (aniseikonia, *p* = 0.46; aniseikonia per aniso-AL 1.00 mm, *p* = 0.74; difference of AL, *p* = 0.74).

The characteristics of the amblyopic and anisometropic groups are summarized in Table 1 and Table 2, respectively. The difference of SE, the difference of AL, and the corneal radius of curvature were comparable between the amblyopic and anisometropic groups (difference of SE, *p* = 0.43; difference of AL, *p* = 0.55; corneal radius of curvature, *p* = 0.26). Visual acuity was significantly different between the amblyopic (0.06 ± 0.07 logMAR) and anisometropic (0.00 ± 0.00 logMAR) groups (*p* = 0.019). Stereoacuity was significantly different between the amblyopic (1.90 ± 0.18 log arcsec) and anisometropic (1.74 ± 0.11 log arcsec) groups (*p* = 0.015).

**Figure 3:**
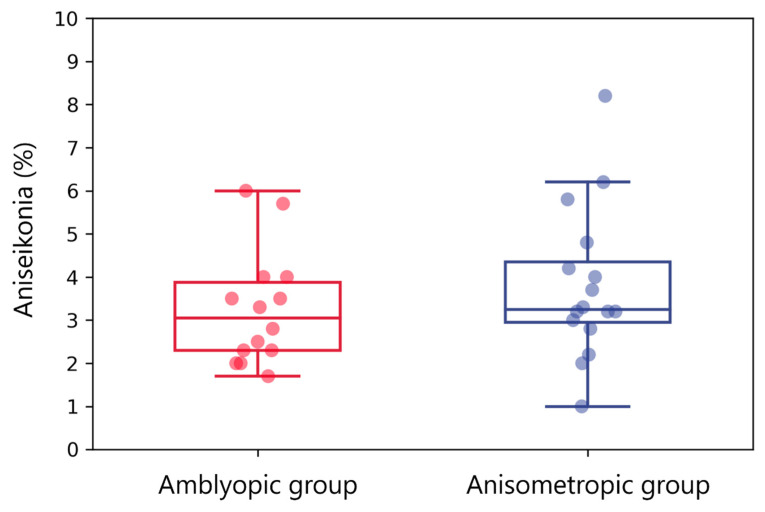
Amount of aniseikonia in the amblyopic and anisometropic groups. Red and blue box plots with dots indicate aniseikonia in the amblyopic and anisometropic groups, respectively. Aniseikonia was insignificantly different between both groups.

The difference in the amount of aniseikonia was insignificant between amblyopic (3.26% ± 1.27% (median, 3.1%; interquartile range (IQR), 2.30–4.00%)) and anisometropic (3.79% ± 1.71% (median, 3.25%; IQR, 2.95–4.50%)) groups (*p* = 0.36; Figure 3).

**Figure 4:**
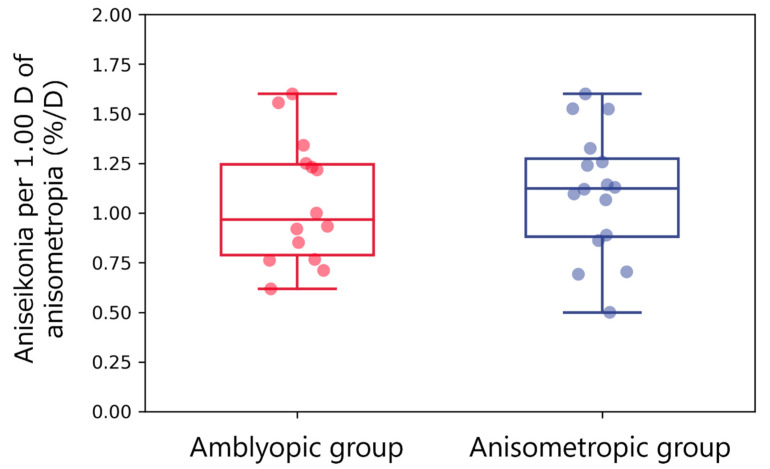
Aniseikonia per 1.00 D of anisometropia. Red and blue box plots with dots show aniseikonia per 1.00 D of anisometropia in the amblyopic and anisometropic groups, respectively. There were insignificant differences between both groups.

The aniseikonia per 1.00 D of anisometropia was comparable between amblyopic (1.05 ± 0.30%/D (median, 0.97%/D; IQR, 0.79–1.23%/D)) and anisometropic (1.10% ± 0.31%/D (median, 1.12%/D; IQR, 0.88–1.29%/D)) groups (*p* = 0.67; Figure 4).

**Figure 5:**
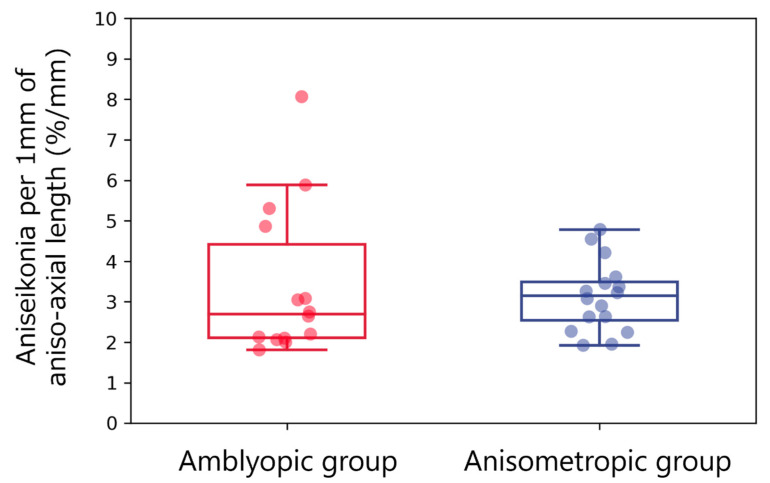
Aniseikonia per 1.00 mm of aniso-axial length. Red and blue box plots with dots indicate aniseikonia per 1.00 mm of aniso-axial length in the amblyopic and anisometropic groups, respectively. There were insignificant differences between both groups.

Aniseikonia per 1.00 mm of aniso-AL was comparable between amblyopic (3.42% ± 1.81%/mm (median, 2.69%/mm; IQR, 2.11–3.08%/mm)) and anisometropic (3.13% ± 0.84%/mm (median, 3.15%/mm; IQR, 2.53–3.53%/mm)) groups (*p* = 0.67; Figure 5). The aniseikonia measurements for each subject in both groups are summarized in Table 3 and Table 4, respectively.

The intrarater ICC (1, 6) was 0.973 (0.943–0.990) for the amblyopic group and 0.982 (0.964–0.998) for the anisometropic group.

**Figure 6:**
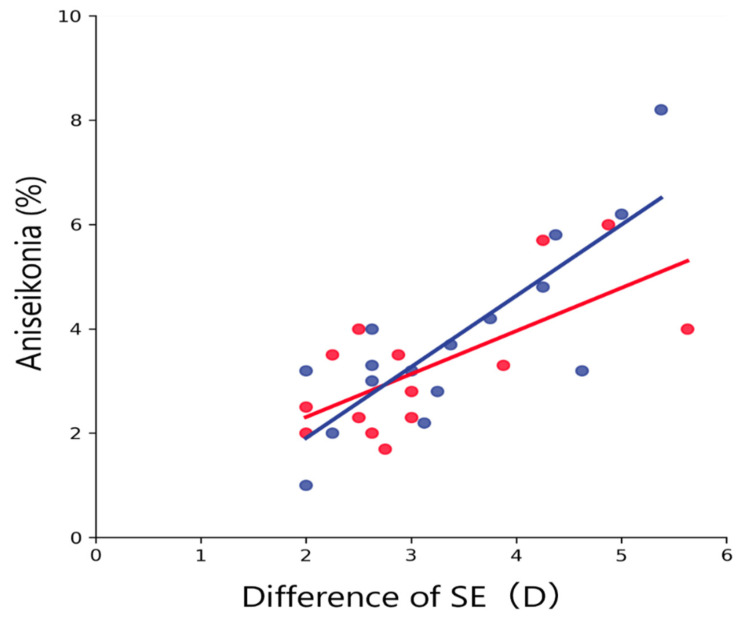
Relationship between aniseikonia and the difference of SE. Red and blue dots indicate the amblyopic and anisometropic groups, respectively. The solid red and blue lines indicate the regression lines for the amblyopic and anisometropic groups, respectively. Both groups showed a significant positive correlation between aniseikonia and the difference of SE. SE, subjective spherical equivalent.

**Figure 7:**
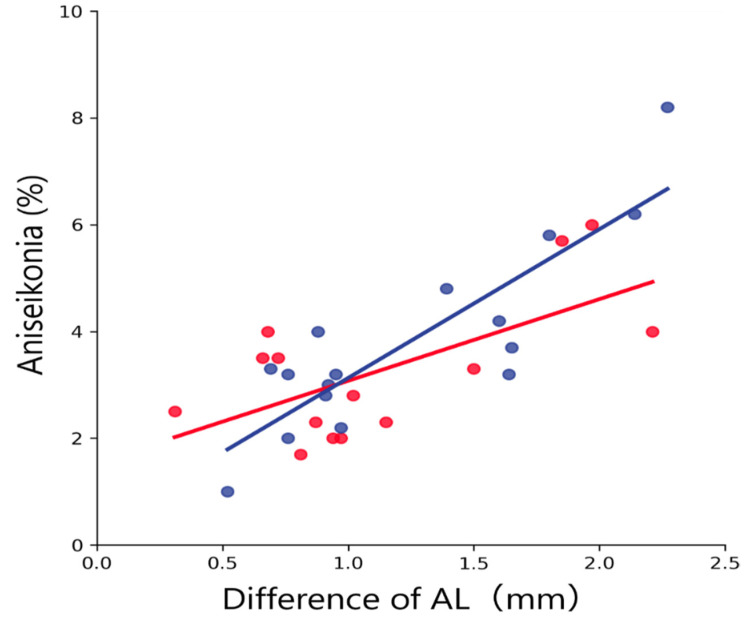
Relationship between aniseikonia and the difference of AL. Red and blue dots indicate the amblyopic and anisometropic groups, respectively. The solid red and blue lines indicate the regression lines for the amblyopic and anisometropic groups, respectively. Both groups showed a significant positive correlation between aniseikonia and the difference of AL. AL, axial length.

**Figure 8:**
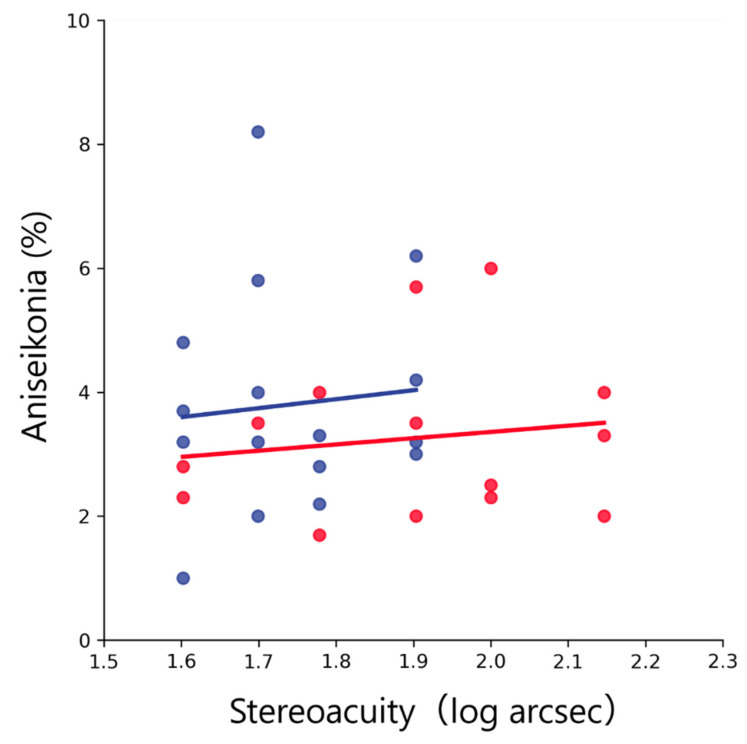
Relationship between aniseikonia and stereoacuity. Red and blue dots indicate the amblyopic and anisometropic groups, respectively. The solid red and blue lines indicate the regression lines for the amblyopic and anisometropic groups, respectively. Both groups showed a not significant correlation between aniseikonia and stereoacuity.

In both groups, aniseikonia was significantly and positively correlated with the difference of SE (amblyopic group, *R*^2^ = 0.47, *p* = 0.007; anisometropic group, *R*^2^ = 0.68, *p* < 0.001; all data merged, *R*^2^ = 0.58, *p* < 0.001; Figure 6) and with the difference of AL (amblyopic group, *R*^2^ = 0.41, *p* = 0.013; anisometropic group, *R*^2^ = 0.74; all data merged, *R*^2^ = 0.59, *p* < 0.001; Figure 7). In both groups, aniseikonia was not significantly correlated with the stereoacuity (amblyopic group, *R*^2^ = 0.020, *p* = 0.95; anisometropic group, *R*^2^ = 0.009, *p* = 0.73; all data merged, *R*^2^ < 0.001, *p* = 0.95; Figure 8).

## 4. Discussion

We compared aniseikonia between the amblyopic and anisometropic groups to evaluate whether aniseikonia is inhibited by amblyopia treatment. The amount of aniseikonia did not differ significantly between the amblyopic and anisometropic groups (Figure 3). Our finding suggests that aniseikonia is not inhibited by amblyopia treatment using fully corrected spectacles even if the patients do not complain of aniseikonic symptoms. Lubkin et al. reported that amblyopia correlated with anisometropia and aniseikonia, and the impact was higher in anisometropia than in aniseikonia [37]. We consider that the association between amblyopia and aniseikonia may be due to the exclusion of confounding variables, because aniseikonia relates to anisometropia. Burian et al. reported that although the symptoms of aniseikonia might be adapted, the amount of aniseikonia remains the same as measured by space eikonometry [8]. Space eikonometry should only use straight lines for the target, to exclude all empirical elements of spatial perception. Therefore, the SAT target also consisted of straight lines exclusively (Figure 2). Therefore, our findings suggest that patients with anisometropic amblyopia hardly complain of symptoms of aniseikonia because of cortical adaptation and suppression, whereas the amount of aniseikonia essentially remains unchanged.

In this study, visual acuity and stereoacuity were significantly lower in the amblyopic group than in the anisometropic group (Table 1 and Table 2). These findings are consistent with earlier studies that concluded that visual acuity, stereoacuity, and contrast sensitivity do not fully recover in patients with anisometropic amblyopia, even if equal to or better than the criteria for amblyopia treatment [38,39,40]. Conversely, Jiménez-Rodríguez et al. reported that four adult patients with anisometropic amblyopia were trained in amblyopia using virtual reality, with good results in visual acuity, contrast sensitivity, and stereoacuity [41]. The amblyopia group included in this study met only the PEDIG recovery criteria for successful amblyopia treatment. Furthermore, the amblyopia group was very young (8.23 ± 1.53 years old). Therefore, it is conceivable that some cases of stereoacuity will improve with age. The longitudinal observation of patients with anisometropic amblyopia remains a subject for future research.

The repeatability for the amount of aniseikonia using the SAT did not significantly differ six times in the amblyopic and anisometropic groups (Table 3 and Table 4). In both groups, the intrarater ICC showed high agreement. This finding is consistent with the results of Sasaki et al., who found that the amount of aniseikonia had a low variation using the SAT [31,32].

The aniseikonia per 1.00 D of anisometropia was not significantly different between both groups. The value was 1.05%/D in the amblyopic group and 1.10%/D in the anisometropic group (Figure 4). This finding supports the optical approximation and empirical rule of 1% aniseikonia per 1.00D of anisometropia [1,42,43,44,45]. In contrast, other earlier studies reported that aniseikonia per 1.00 D of anisometropia was less than 1.0%/D [20,46]. These earlier studies evaluated aniseikonia using direct comparison methods, such as New Aniseikonia Tests (HANDAYA Inc., Tokyo, Japan) and Aniseikonia Inspector (Optical Diagnostics, Culemborg, The Netherlands). The direct comparison method underestimates aniseikonia because of the influence of peripheral fusion and sensory adaptation to cancel out image differences caused by aniseikonia in binocular vision [24,25,26,27]. South et al. showed that the amount of aniseikonia in the patients with anisometropic amblyopia had significant variation [20]. Moreover, El-Abedin Rajab et al. reported that the direct comparison method in axial anisometropia did not produce indications of significant aniseikonia [47]. Thus, aniseikonia in anisometropic amblyopia using the direct comparison method varies from the report.

The aniseikonia per 1.00 D of aniso-AL was not significantly different between both groups, and that value was 3.42%/D in the amblyopic group and 3.13%/D in the anisometropic group. We consider this result accurate because AL strongly correlates with ocular refraction. The ocular refraction per 1.0 mm of AL ranges between 2.00 and 3.00 D [48,49,50,51,52].

Aniseikonia was significantly and positively correlated with the differences of SE and AL within both groups (Figure 6 and Figure 7). These findings are consistent with the earlier studies evaluating aniseikonia using direct comparison methods [20]. In earlier studies, the direct comparison methods Aniseikonia Inspector and New Aniseikonia Tests were used to measure aniseikonia for anisometropic amblyopia. The method stated that although there was significant variability between tests, a significant trend was observed for Aniseikonia Inspector (*R* = 0.63, *R*^2^ = 0.40, *p* = 0.005) and New Aniseikonia Tests (*R* = 0.54, *R*^2^ = 0.29, *p* = 0.017), with subjective aniseikonia increasing as disparity increased. Compared to the current results, the correlation was higher for the SAT. However, many of those subjects studied by South et al. had poor stereopsis [20]. In addition, the number of subjects was smaller than ours, and a control group was included in addition to the anisometropic amblyopia group when determining correlations. Therefore, although there was a trend toward an increase in aniseikonia with an increase in anisometropia, as observed in the direct comparison method, it is difficult to compare these findings with our results.

Axial anisometropia was more common in this study because it included SE differences of ≥2.00 D, and no significant difference in the radius of the corneal curvature was observed between the left and right eyes. The results of this study are supported by those reported by Sorsby et al. [53], who reported that anisometropia above 2.00 D had a high rate of axial anisometropia. These results suggest that aniseikonia is also common in anisometropic amblyopia and that aniseikonia increases as the difference of SE and AL increases.

Aniseikonia was not significantly correlated with the stereoacuity (Figure 8). Earlier studies have reported that stereopsis decreases curvilinearly as aniseikonia increases [11]. We have considered that the range of amount of aniseikonia is different between the present and earlier studies. Although the present evaluated aniseikonia was between 1% and 8.2%, earlier studies evaluated aniseikonia between 1.2% and 32.3% using the placing focal magnifiers. Furthermore, in this study, we defined the amblyopic group as patients who had successful amblyopia treatment. Therefore, our finding suggests that in anisometropic amblyopia with successful amblyopia treatment, stereoacuity is not necessarily reduced even if aniseikonia is large.

This study showed that aniseikonia remained even after successful treatment of anisometropic amblyopia, and the amounts of aniseikonia were not different between the amblyopic and anisometropic groups. Thus, we plan to evaluate the change of amount of aniseikonia in a long-term observation. A limitation of this study is the relatively narrow range of inequality of vision evaluated. This may limit the evaluation of the full range of aniseikonia after amblyopia treatment. In addition, we did not evaluate aniseikonia in patients with anisometropic amblyopia during amblyopia treatment. We plan to investigate changes in aniseikonia observed at the initial visit during amblyopia treatment. Additionally, there were more cases of hyperopic anisometropia in the amblyopic group and myopic anisometropia in the anisometropic group. Age and the nature of anisometropia (hyperopia or myopia) are limitations of this study. The earlier studies reported that anisometropic amblyopia is more common in hyperopic anisometropia [54,55]. Therefore, future studies should increase the sample size, match the ages, and examine hyperopic and myopic anisometropia separately.

## 5. Conclusions

Aniseikonia in patients with anisometropic amblyopia is not inhibited after complete amblyopia treatment, even if the patients did not complain of aniseikonic symptoms. The amount of aniseikonia is similar between the amblyopic and anisometropic groups.

## 6. Patents

Kakeru Sasaki, JP-A-2016-52464.

## Figures and Tables

**Table 1:** Participants’ characteristics in the amblyopic group.

			Visual Acuity (logMAR)										
ID	Age (y)	Duration of Amblyopia Treatment (Month)	Initiation of Treatment		Current		SE (D)		Corneal Radius of Curvature (mm)		AL (mm)		Stereoacuity (log arcsec)
			RE	LE	RE	LE	RE	LE	RE	LE	RE	LE	
1	10	60	−0.08	0.40	−0.08	−0.08	0.00	2.00	8.36	8.41	24.22	23.91	2.00
2	13	119	0.52	0.00	−0.08	−0.08	5.63	0.00	7.68	7.62	21.18	23.39	2.15
3	10	30	0.00	0.15	−0.18	−0.08	1.00	4.00	7.76	7.72	22.27	21.12	1.60
4	8	13	0.52	−0.08	−0.08	−0.08	4.88	2.00	7.99	7.77	21.42	22.14	1.70
5	8	21	−0.08	0.22	−0.08	−0.08	2.25	6.50	8.07	8.03	22.56	20.71	1.90
6	9	27	−0.08	0.15	−0.08	0.10	3.88	6.38	7.85	7.90	21.58	20.90	1.78
7	8	41	−0.08	0.52	−0.08	0.00	−0.50	2.13	8.00	8.10	23.58	22.61	1.90
8	6	40	0.00	1.05	−0.08	0.05	1.00	4.88	7.78	7.78	22.39	20.89	2.15
9	6	39	0.52	0.10	0.00	−0.08	4.75	2.50	7.83	7.82	21.10	21.76	1.90
10	6	36	0.70	0.00	−0.08	−0.08	5.00	2.00	7.62	7.56	20.57	21.59	1.60
11	9	8	0.40	−0.08	0.10	−0.08	2.75	0.00	7.80	7.77	22.60	23.41	1.78
12	8	18	−0.08	0.30	−0.08	−0.08	1.50	4.00	7.72	7.75	22.15	21.28	2.00
13	11	94	0.22	1.10	−0.30	0.05	0.00	4.88	7.97	8.13	23.56	21.59	2.00
14	8	55	0.40	−0.08	0.00	−0.08	3.25	1.25	7.81	7.86	21.74	22.68	2.15

y, years; RE, right eye; LE, left eye; logMAR, logarithm of minimum angle of resolution; SE, subjective spherical equivalent; D, diopter; AL, axial length; log arcsec, logarithm of arcsecond.

**Table 2:** Participants’ characteristics in the anisometropic group.

ID	Age (y)	Visual Acuity (logMAR)		SE (D)		Corneal Radius of Curvature (mm)		AL (mm)		Stereoacuity (log arcsec)
		RE	LE	RE	LE	RE	LE	RE	LE	
1	20	−0.08	−0.08	2.63	−0.50	7.71	7.67	21.75	22.72	1.78
2	21	−0.08	−0.08	0.75	−1.88	7.27	7.30	22.70	23.62	1.90
3	20	−0.08	−0.08	0.50	−1.75	7.85	7.82	23.84	24.60	1.70
4	19	−0.08	−0.08	1.50	3.50	7.52	7.51	22.38	21.86	1.60
5	19	−0.08	−0.08	−5.00	−9.25	7.65	7.61	25.43	26.82	1.60
6	19	−0.08	−0.08	−2.25	−0.25	7.51	7.47	24.00	23.24	1.90
7	19	−0.08	−0.08	−3.13	−5.75	7.73	7.64	24.89	25.58	1.78
8	20	−0.08	−0.08	−0.25	−5.25	7.58	7.57	23.11	25.25	1.90
9	24	−0.08	−0.08	3.75	−0.63	7.84	7.92	22.41	24.21	1.70
10	20	−0.08	−0.08	−8.38	−5.13	7.15	7.18	24.99	24.08	1.78
11	22	−0.08	−0.08	−4.50	−0.75	7.89	7.83	26.78	25.18	1.90
12	21	−0.08	−0.08	−4.75	−2.13	7.42	7.43	24.61	23.73	1.70
13	24	−0.08	−0.08	−2.00	1.00	7.56	7.54	23.23	22.28	1.60
14	19	−0.08	−0.08	−5.75	−0.38	8.08	8.02	26.29	24.02	1.70
15	19	−0.08	−0.08	−4.00	−0.63	8.01	8.07	26.03	24.38	1.60
16	19	−0.08	−0.08	−4.63	0.00	7.86	7.88	25.08	23.44	1.70

y, years; RE, right eye; LE, left eye; logMAR, logarithm of minimum angle of resolution; SE, subjective spherical equivalent; D, diopter; AL, axial length; log arcsec, logarithm of arcsecond.

**Table 3:** Measurements of aniseikonia in the amblyopic group.

ID	Aniseikonia (%)								Aniseikonia per 1.00 D of Anisometropia (%/D)	Aniseikonia per 1 mm of Aniso-Axial Length (%/mm)
	1st	2nd	3rd	4th	5th	6th	Mean	SD		
1	3	2	3	2	3	2	2.5	0.5	1.3	8.1
2	3	4	4	4	5	4	4	0.6	0.7	1.8
3	3	2	3	2	2	2	2.3	0.5	0.8	2.0
4	3	4	3	3	4	4	3.5	0.5	1.2	4.9
5	6	6	5	5	5	7	5.7	0.7	1.3	3.1
6	4	5	3	4	4	4	4	0.6	1.6	5.9
7	2	2	2	2	2	2	2	0.0	0.8	2.1
8	4	3	3	3	4	3	3.3	0.5	0.9	2.2
9	4	4	3	3	3	4	3.5	0.5	1.6	5.3
10	2	3	3	3	3	3	2.8	0.4	0.9	2.7
11	2	1	2	2	1	2	1.7	0.5	0.6	2.1
12	2	3	2	2	3	2	2.3	0.5	0.9	2.6
13	6	6	5	6	7	6	6	0.6	1.2	3.0
14	2	2	2	2	2	2	2	0.0	1.0	2.1

SD, standard deviation.

**Table 4:** Measurements of aniseikonia in the anisometropic group.

ID	Aniseikonia (%)								Aniseikonia per 1.00 D of Anisometropia (%/D)	Aniseikonia per 1 mm of Aniso-Axial Length (%/mm)
	1st	2nd	3rd	4th	5th	6th	Mean	SD		
1	2	1	2	2	3	3	2.2	0.7	0.7	2.3
2	3	3	3	3	3	3	3	0.0	1.1	3.3
3	2	2	2	2	2	2	2	0.0	0.9	2.6
4	1	1	1	1	1	1	1	0.0	0.5	1.9
5	5	5	4	5	5	5	4.8	0.4	1.1	3.5
6	3	3	3	3	3	4	3.2	0.4	1.6	4.2
7	4	3	3	4	3	3	3.3	0.5	1.3	4.8
8	7	6	5	7	6	6	6.2	0.7	1.2	2.9
9	5	5	6	7	5	7	5.8	0.9	1.3	3.2
10	3	3	3	2	3	3	2.8	0.4	0.9	3.1
11	5	4	4	4	4	4	4.2	0.4	1.1	2.6
12	4	4	4	4	4	4	4	0.0	1.5	4.5
13	2	3	2	4	4	4	3.2	0.9	1.1	3.4
14	8	10	8	7	8	8	8.2	0.9	1.5	3.6
15	4	3	4	4	4	3	3.7	0.5	1.1	2.2
16	3	3	4	3	3	3	3.2	0.4	0.7	2.0

SD, standard deviation.

## Data Availability

All relevant data are available within the manuscript.

## References

[1] Ogle K.N. (1950). Researches in Binocular Vision.

[2] Furr B.A. (2019). Aniseikonia: A 21st Century Look. J. Binocul. Vis. Ocul. Motil..

[3] South J., Gao T., Collins A., Turuwhenua J., Robertson K., Black J. (2019). Aniseikonia and anisometropia: Implications for suppression and amblyopia. Clin. Exp. Optom..

[4] Bannon R.E., Triller W. (1944). Aniseikonia—A Clinical Report Covering a Ten Year Period. Clin. Exp. Optom..

[5] Berens C., Loutfallah M. (1938). Aniseikonia: A Study of 836 Patients Examined with the Ophthalmo-Eikonometer. Trans. Am. Ophthalmol. Soc..

[6] Flom M.C., Bedell H.E. (1985). Identifying amblyopia using associated conditions, acuity, and nonacuity features. Am. J. Optom. Physiol. Opt..

[7] Burian H.M. (1943). Clinical Significance of Aniseikonia. Arch. Ophthalmol..

[8] Burian H.M. (1943). Influence Of Prolonged Wearing of Meridional Size Lenses on Spatial Localization. Arch. Ophthalmol..

[9] Lovasik J.V., Szymkiw M. (1985). Effects of aniseikonia, anisometropia, accommodation, retinal illuminance, and pupil size on stereopsis. Investig. Ophthalmol. Vis. Sci..

[10] Katsumi O., Tanino T., Hirose T. (1986). Effect of aniseikonia on binocular function. Investig. Ophthalmol. Vis. Sci..

[11] Atchison D.A., Nguyen T., Schmid K.L., Rakshit A., Baldwin A.S., Hess R.F. (2022). The effects of optically and digitally simulated aniseikonia on stereopsis. Ophthalmic Physiol. Opt..

[12] Reed B. (2011). Aniseikonia: A Case Series and Literature Review.

[13] Okamoto F., Sugiura Y., Okamoto Y., Hiraoka T., Oshika T. (2017). Aniseikonia in various retinal disorders. Graefe’s Arch. Clin. Exp. Ophthalmol..

[14] Okamoto F., Morikawa S., Sugiura Y., Hoshi S., Hiraoka T., Oshika T. (2020). Preoperative aniseikonia is a prognostic factor for postoperative stereopsis in patients with unilateral epiretinal membrane. Graefe’s Arch. Clin. Exp. Ophthalmol..

[15] Okamoto F., Sugiura Y., Okamoto Y., Hiraoka T., Oshika T. (2014). Aniseikonia and foveal microstructure after retinal detachment surgery. Investig. Ophthalmol. Vis. Sci..

[16] Okamoto F., Sugiura Y., Okamoto Y., Hiraoka T., Oshika T. (2014). Time course of changes in aniseikonia and foveal microstructure after vitrectomy for epiretinal membrane. Ophthalmology.

[17] Bradley A., Rabin J., Freeman R.D. (1983). Nonoptical determinants of aniseikonia. Investig. Ophthalmol. Vis. Sci..

[18] Weakley D.R. (2001). The association between nonstrabismic anisometropia, amblyopia, and subnormal binocularity. Ophthalmology.

[19] Cotter S.A., Edwards A.R., Wallace D.K., Beck R.W., Arnold R.W., Astle W.F., Barnhardt C.N., Birch E.E., Donahue S.P., Everett D.F. (2006). Treatment of anisometropic amblyopia in children with refractive correction. Ophthalmology.

[20] South J., Gao T., Collins A., Lee A., Turuwhenua J., Black J. (2020). Clinical aniseikonia in anisometropia and amblyopia. Br. Ir. Orthopt. J..

[21] Awaya S., Sugawara M., Horibe F., Torii F. (1982). [The “new aniseikonia tests” and its clinical applications (author’s transl)]. Nippon Ganka Gakkai Zasshi.

[22] Corliss D.A., Rutstein R.P., Than T.P., Hopkins K.B., Edwards C. (2005). Aniseikonia testing in an adult population using a new computerized test, “the Aniseikonia Inspector”. Binocul. Vis. Strabismus Q..

[23] Kehler L.A.F., Fraine L., Lu P. (2014). Evaluation of the Aniseikonia Inspector Version 3 in School-Aged Children. Optom. Vis. Sci..

[24] McCormack G., Peli E., Stone P. (1992). Differences in tests of aniseikonia. Investig. Ophthalmol. Vis. Sci..

[25] Rutstein R.P., Corliss D.A., Fullard R.J. (2006). Comparison of aniseikonia as measured by the aniseikonia inspector and the space eikonometer. Optom. Vis. Sci..

[26] Antona B., Barra F., Barrio A., Gonzalez E., Sanchez I. (2007). Validity and repeatability of a new test for aniseikonia. Investig. Ophthalmol. Vis. Sci..

[27] Antona B., Barra F., Barrio A., Gonzalez E., Sánchez I. (2006). The Validity and Repeatability of the New Aniseikonia Test. Optom. Vis. Sci..

[28] Bannon R.E. (1953). Space eikonometry in aniseikonia. Am. J. Optom. Arch. Am. Acad. Optom..

[29] Ames A. (1945). The Space Eikonometer Test for Aniseikonia*. Am. J. Ophthalmol..

[30] Ogle K.N. (1946). Theory of the Space-Eikonometer. J. Opt. Soc. Am..

[31] Sasaki K., Kobayashi K., Usui C., Hayashi T., Kawashima M., Tane Y., Mizota A. (2017). Evaluation of newly-developed aniseikonia testing method based on space eikonometry. Clin. Exp. Optom..

[32] Sasaki K., Kida J., Kobayashi K. (2017). New Aniseikonia Testing Method based on Space Eikonometry. Jpn. J. Vis. Sci..

[33] Repka M.X., Kraker R.T., Beck R.W., Holmes J.M., Cotter S.A., Birch E.E., Astle W.F., Chandler D.L., Felius J., Arnold R.W. (2008). A randomized trial of atropine vs. patching for treatment of moderate amblyopia: Follow-up at age 10 years. Arch. Ophthalmol..

[34] Scheiman M.M., Hertle R.W., Kraker R.T., Beck R.W., Birch E.E., Felius J., Holmes J.M., Kundart J., Morrison D.G., Repka M.X. (2008). Patching vs. atropine to treat amblyopia in children aged 7 to 12 years: A randomized trial. Arch. Ophthalmol..

[35] Jia Y., Liu J., Ye Q., Zhang S., Feng L., Xu Z., Zhuang Y., He Y., Zhou Y., Chen X. (2022). Factors predicting regression of visual acuity following successful treatment of anisometropic amblyopia. Front. Med..

[36] Group T.P.E.D.I. (2002). A Randomized Trial of Atropine vs Patching for Treatment of Moderate Amblyopia in Children. Arch. Ophthalmol..

[37] Lubkin V., Kramer P., Meininger D., Shippman S., Bennett G., Visintainer P. (1999). Aniseikonia in relation to strabismus, anisometropia and amblyopia. Binocul. Vis. Strabismus Q..

[38] Wallace D.K., Lazar E.L., Melia M., Birch E.E., Holmes J.M., Hopkins K.B., Kraker R.T., Kulp M.T., Pang Y., Repka M.X. (2011). Stereoacuity in children with anisometropic amblyopia. J. AAPOS.

[39] Wang G., Zhao C., Ding Q., Wang P. (2017). An Assessment of the Contrast Sensitivity in Patients with Ametropic and Anisometropic Amblyopia in Achieving the Corrected Visual Acuity of 1.0. Sci. Rep..

[40] Jia Y., Ye Q., Zhang S., Feng L., Liu J., Xu Z., Zhuang Y., He Y., Zhou Y., Chen X. (2022). Contrast Sensitivity and Stereoacuity in Successfully Treated Refractive Amblyopia. Investig. Ophthalmol. Vis. Sci..

[41] Jiménez-Rodríguez C., Yélamos-Capel L., Salvestrini P., Pérez-Fernández C., Sánchez-Santed F., Nieto-Escámez F. (2023). Rehabilitation of visual functions in adult amblyopic patients with a virtual reality videogame: A case series. Virtual Real..

[42] Lubkin V., Shippman S., Bennett G., Meininger D., Kramer P., Poppinga P. (1999). Aniseikonia quantification: Error rate of rule of thumb estimation. Binocul. Vis. Strabismus Q..

[43] Berens C., Bannon R.E. (1963). Aniseikonia A Present Appraisal and Some Practical Considerations. Arch. Ophthalmol..

[44] Linksz A., Bannon R.E. (1965). Aniseikonia and Refractive Problems. Int. Ophthalmol. Clin..

[45] Achiron L.R., Witkin N., Primo S., Broocker G. (1997). Contemporary management of aniseikonia. Surv. Ophthalmol..

[46] Tayah D., Tannous M., Tayah Y.S., Alves M.R. (2022). Association between Anisometropia as Well as Visual Acuity, Aniseikonia, and Stereopsis in the Absence of Strabismus. Open J. Ophthalmol..

[47] El-Abedin Rajab G., Elaziz M., Soliman S., Basiony A. (2022). Measurement of spectacle-induced aniseikonia in axial anisometropia using the New Aniseikonia Test. J. Egypt. Ophthalmol. Soc..

[48] Fledelius H.C. (1982). Ophthalmic Changes from Age of 10 to 18 Years. Acta Ophthalmol..

[49] Saw S.M., Chua W.H., Gazzard G., Koh D., Tan D.T.H., Stone R.A. (2005). Eye growth changes in myopic children in Singapore. Br. J. Ophthalmol..

[50] Cruickshank F.E., Logan N.S. (2018). Optical ‘dampening’ of the refractive error to axial length ratio: Implications for outcome measures in myopia control studies. Ophthalmic Physiol. Opt..

[51] Atchison D.A., Jones C.E., Schmid K.L., Pritchard N., Pope J.M., Strugnell W.E., Riley R.A. (2004). Eye Shape in Emmetropia and Myopia. Investig. Ophthalmol. Vis. Sci..

[52] Wong H.-B., Machin D., Tan S.-B., Wong T.-Y., Saw S.-M. (2010). Ocular Component Growth Curves among Singaporean Children with Different Refractive Error Status. Investig. Ophthalmol. Vis. Sci..

[53] Sorsby A., Leary G.A., Joan Richards M. (1962). The optical components in anisometropia. Vis. Res..

[54] Jampolsky A., Flom B.C., Weymouth F.W., Moses L.E. (1955). Unequal Corrected Visual Acuity as Related to Anisometropia. AMA Arch. Ophthalmol..

[55] Akbarzadeh S., Vahabi R., Bazzazi N., Roshanaei G., Heydarian S., Fouladi D.F. (2018). The burden of pure anisometropic amblyopia: A cross-sectional study on 2800 Iranians. Int. Ophthalmol..

